# Sex-linked genomic variation and its relationship to avian plumage dichromatism and sexual selection

**DOI:** 10.1186/s12862-015-0480-4

**Published:** 2015-09-16

**Authors:** Huateng Huang, Daniel L. Rabosky

**Affiliations:** Department of Ecology and Evolutionary Biology and Museum of Zoology, University of Michigan, Ann Arbor, MI 48109 USA

## Abstract

**Background:**

Sexual dichromatism is the tendency for sexes to differ in color pattern and represents a striking form of within-species morphological variation. Conspicuous intersexual differences in avian plumage are generally thought to result from Darwinian sexual selection, to the extent that dichromatism is often treated as a surrogate for the intensity of sexual selection in phylogenetic comparative studies. Intense sexual selection is predicted to leave a footprint on genetic evolution by reducing the relative genetic diversity on sex chromosome to that on the autosomes.

**Results:**

In this study, we test the association between plumage dichromatism and sex-linked genetic diversity using eight species pairs with contrasting levels of dichromatism. We estimated Z-linked and autosomal genetic diversity for these non-model avian species using restriction-site associated (RAD) loci that covered ~3 % of the genome. We find that monochromatic birds consistently have reduced sex-linked genomic variation relative to phylogenetically-paired dichromatic species and this pattern is robust to mutational biases.

**Conclusions:**

Our results are consistent with several interpretations. If present-day sexual selection is stronger in dichromatic birds, our results suggest that its impact on sex-linked genomic variation is offset by other processes that lead to proportionately lower Z-linked variation in monochromatic species. We discuss possible factors that may contribute to this discrepancy between phenotypes and genomic variation. Conversely, it is possible that present-day sexual selection -- as measured by the variance in male reproductive success -- is stronger in the set of monochromatic taxa we have examined, potentially reflecting the importance of song, behavior and other non-plumage associated traits as targets of sexual selection. This counterintuitive finding suggests that the relationship between genomic variation and sexual selection is complex and highlights the need for a more comprehensive survey of genomic variation in avian taxa that vary markedly in social and genetic mating systems.

**Electronic supplementary material:**

The online version of this article (doi:10.1186/s12862-015-0480-4) contains supplementary material, which is available to authorized users.

## Background

Biologists have generally assumed that sexual selection drives the evolution of sexual dimorphism, as proposed by Darwin [1] and it is now clear that many secondary sexual traits are targets of sexual selection [2]. The striking extent of plumage dichromatism in birds is considered a textbook example of sexual selection and sexes of some species are so dissimilar that they were initially considered to represent distinct species [2]. Alfred Russell Wallace suggested that the drab colors of (usually) female birds might reflect the need for cryptic coloration during nesting [3], and this hypothesis has received some support in recent years [4, 5]. However, the general assumption is that sexual dichromatism — with males typically more conspicuous than females — is maintained by ongoing sexual selection, either through female preference or advantages in male-male competition. Numerous field observation and manipulative experiments have shown that female birds prefer brighter males [6] and male coloration frequently correlates with reproductive success [7]. The correlation between plumage dichromatism and social mating system in birds [8, 9] is another piece of evidence often cited to support the use of dichromatism as a surrogate for sexual selection intensity in comparative studies [10].

While there is now extensive evidence that plumage-associated traits are the targets of sexual selection, the general assumption that species with greater intersexual plumage differences experience stronger sexual selection remains largely untested. The fact that many highly dichromatic bird species are socially monogamous and the existence of widespread extra-pair paternity [11] suggests possible disconnects among dichromatism, social mating system and sexual selection. Moreover, evidence that plumage traits with intersexual differences experience ongoing sexual selection within species does not necessarily indicate reduced selection in species that lack dichromatic plumage. Song complexity, for example, could be an alternative target for sexual selection in bird species [12].

One direct measurement of sexual selection that is comparable between species is the variance in reproductive success among the sex with lower parental investment in offspring [13–15]. While measuring individual mating success is non-trivial for most wild bird populations, an index from population genetics—the relative levels of genetic diversity of the sex chromosomes to that of the autosomes—could be informative about the reproductive variance [16, 17]. For most avian species, stronger sexual selection is expected to increase the variance in male reproductive success relative to females. As male birds have two copies of the Z chromosome versus one in females, reduction in the effective size of male breeding populations caused by sexual selection would reduce the effective population size of the Z chromosome more dramatically than that the autosomes [18]. We thus expect that the genomic footprint of sexual selection could be captured by the ratio of effective population sizes of Z chromosomes and autosomes (*R*_*Z:A*_), which can be calculated from neutral genetic diversity estimates (i.e., *θ = 4Nμ*; where *μ* is the mutation rate and *N* is the effective population size). That is, if dichromatic species are experiencing stronger sexual selection, their *R*_*Z:A*_ should be lower compared to monochromatic species.

We developed an approach to estimate *R*_*Z:A*_ using restriction-site associated DNA RAD; [19] markers sequenced on the Illumina platform. This approach takes advantage of the large number of independent loci generated by next-generation sequencing, which could provided accurate estimates of genetic diversity even from one or several individuals i.e., basing on the heterozygosity of one individual; [20, 21]. We used this method to test the association between *R*_*Z:A*_ and plumage dichromatism across a set of phylogenetically-paired species of North American birds.

## Results

We studied eight matching species pairs that are characterized by contrasting patterns of dichromatism, comprising seven avian families (Additional file 1: Table S1). The focal species included a number of common North American birds, such as the Red-Winged Blackbird (*Agelaius phoeniceus*), Eastern Bluebird (*Sialia sialis*), and Eastern Meadowlark (*Sturnella magna*). After filtering and quality controls (see Methods for details), we obtained an average of 40 Mbps alignment (excluding gaps) between RAD loci and the zebra finch genome, or ~3.3 % of the genome (Additional file 2: Figure S1). Our approach was sufficiently data-rich that *R*_*Z:A*_ could be robustly estimated from heterozygosity information within single individuals (Additional file 3: Figure S2).

If stronger sexual selection leads to increased sperm production (i.e., more cell divisions in males), genetic diversity on the Z chromosome (*θ*_*Z*_) might increase simply because of elevated mutation rates for Z-linked loci (*μ*_*Z*_, as *θ*_*Z*_ = *4N*_*Z*_*μ*_*Z*_). This is a potential problem for our test, as the difference between species in *R*_*Z:A*_ might reflect the difference in mutation rates rather than the effective sizes of breeding population. To correct for this potential male mutational bias, we estimated *R*_*Z:A*_ as the ratio of effective population size (*N*_*Z*_*/N*_*A*_), which is the ratio of genetic diversity (*θ*_*Z*_*/θ*_*A*_) divided by the ratio of mutation rate (*μ*_*Z*_/*μ*_*A*_). We estimated the substitution rates (*μ*) from the divergence between RAD sequences and zebra finch genomes. As expected, we observed male mutation biases as previous studies—the substitution rate is 1.13 times higher for Z-linked loci estimates are 1.08 and 1.10 from [22, 23], respectively. Yet, dichromatic species do not show elevated mutation bias (Additional file 4: Figure S3; see Additional file 5: Figure S4 for similar pattern with different data filtering criterions and estimation methods of *μ*), and the overall pattern does not change (Additional file 3: Figure S2).

**Fig. 1:**
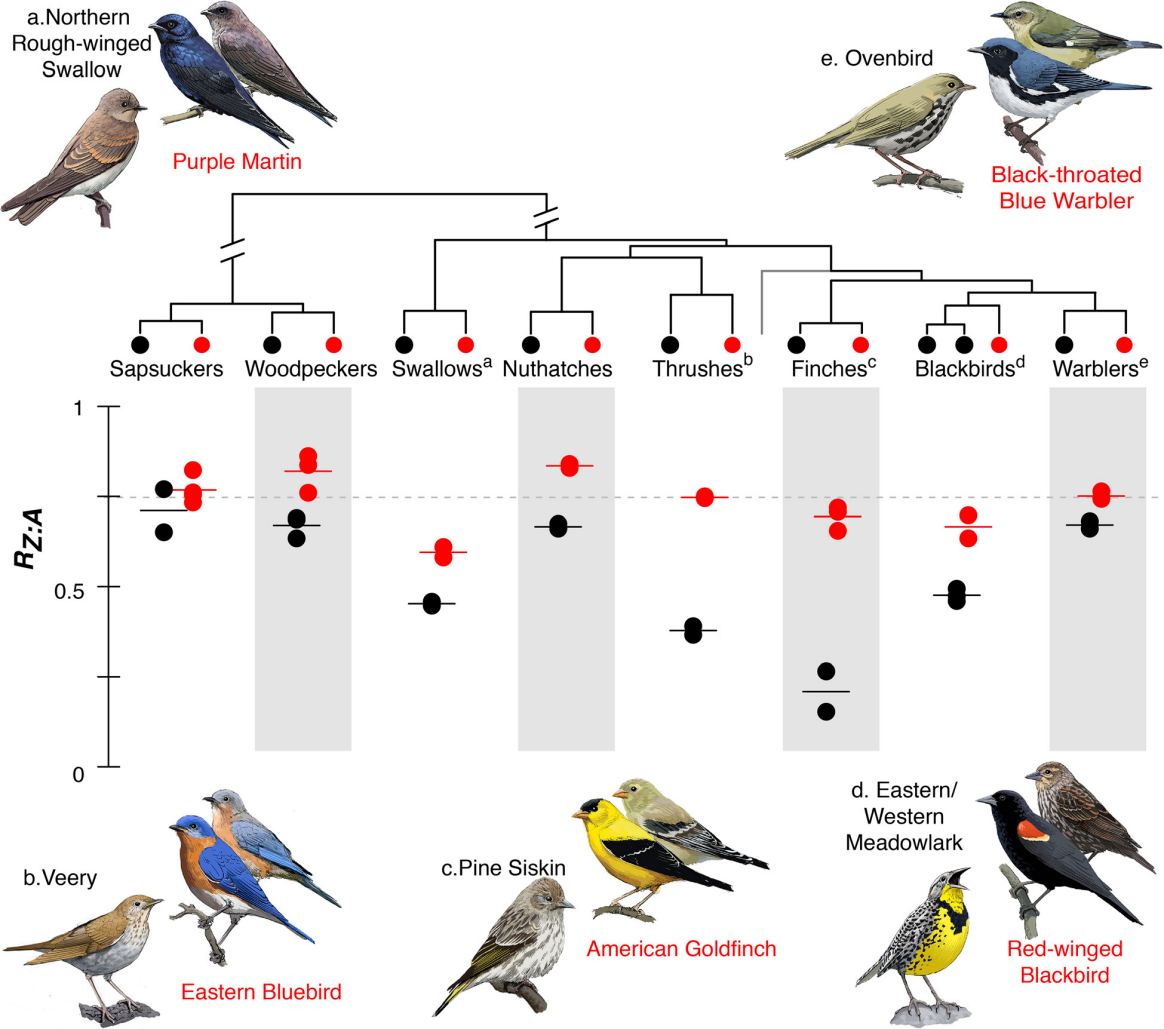
Ratios of Z-associated and autosomal effective population sizes. Matched pairs of sexually dichromatic (red dots) and monochromatic (black dots) bird species are plotted together. Short horizontal lines represent species averages across individuals. The top panel shows the phylogenetic relationship among studied species (grey line represents the position of Zebra Finch). Five of the species pairs are illustrated in surrounding plates with upper right dichromatic species (males in front) and lower left monochromatic species. The three pairs not shown are: Williams’ Sapsucker vs. Red-breasted Sapsucker, Red-bellied Woodpecker vs. Red-headed Woodpecker, and Red-breasted Nuthatch vs. Pygmy Nuthatch. Monochromatic species have reduced genetic variation on the Z chromosome compared to its matching dichromatic species (Wilcoxon test *p* = 0.008; mixed-effect linear regression *p* < 0.001; PGLS regression *p* < 0.001; see Additional file 6 for details). Grey dotted line represents the null expectation that Z-associated effective population size is ¾ of autosomal effective population size

**Fig. 2:**
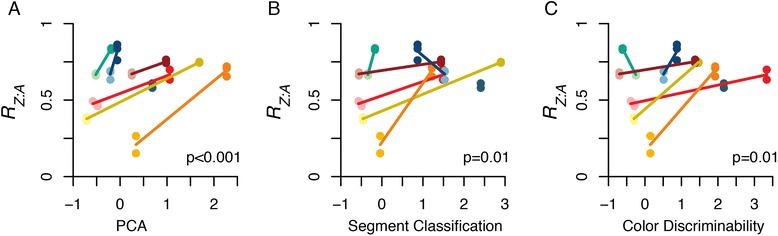
The correlation between reflectance-based dichromatism scores and *R*_*Z:A*_. Three different quantitative measurements of dichromatism —(**a**) PCA, (**b**) segment classification and (**c**) color discriminability—were obtained from published dataset in [24]. Light and dark paired colors represent dichromatic and monochromatic species pairs (coded in the same colors as Fig. 1). If both species in a pair have quantitative measurements of dichromatism available, their species means are connected by a solid line. Two pairs show reversed level of dichromatism in one of the three indices: red-headed woodpecker has a higher segment classification than red-bellied woodpecker (b, dark blue line), and pygmy nuthatcher has a higher color discriminability than red-breasted nuthacher (c, green line). It is unknown whether these are due to possible dichromatism outside of human visual spectrum or measurement errors. Yet, the overall correlations are still significant (*p* values from PGLS regression are shown in lower right corner)

We found that estimates of *R*_*Z:A*_ are highly correlated among individuals of the same species, and *R*_*Z:A*_ was consistently lower in monochromatic species relative to dichromatic species (Fig. 1). Because some components of plumage dichromatism occur outside of the human-visible color spectrum (e.g., ultraviolet), some monochromatic species in human vision might be actually dichromatic. Hence, we repeated our analyses using quantitative reflectance-based dichromatism scores published previously [24] and found a similar negative correlation (Fig. 2).

## Discussion

In this study, we investigated the association between plumage dichromatism and genomic variation in birds. Under the general assumption that greater dichromatism level reflects stronger sexual selection, dichromatic species should have reduced genetic diversity on Z chromosome. We discovered an unexpected yet unequivocal pattern of reduced Z-linked genetic diversity in monochromatic species as compared to phylogenetically-paired dichromatic species (Fig. 1). A simple interpretation of this pattern is that the present-day intensity of sexual selection is lower in the dichromatic species we have studied relative to monochromatic species.

However, as we discuss below, *R*_*Z:A*_ estimates can be influenced by many factors other than sexual selection [16]. Likewise, sexual dichromatism reflects past evolutionary history as well as current ecological conditions [25]. In the following discussion, we examine potential causes of the discrepancy between phenotype and genetic diversity on three different levels. We first consider statistical biases, such that estimates of *R*_*Z:A*_ differ from the true *R*_*Z:A*_. Second, we examine factors that can influence *R*_*Z:A*_ that are potentially unrelated to dichromatism. Third, we consider life history and other traits that are known to correlate with dichromatism, and we explain how they might affect *R*_*Z:A*_.

We emphasize that most confounding factors of *R*_*Z:A*_ cannot fully explain our results, because our focus has been on the relative differences between contrasting species pairs and not on the absolute value of *R*_*Z:A*_. Moreover, most of these confounding factors are expected to add noise to our test: they would suffice to explain a lack of correlation between *R*_*Z:A*_ and dichromatism, but not to generate the consistently negative correlation we observed in our study. By examining factors that are known to associate with genetic diversity and dichromatism phenotypes, we discuss a few possible mechanisms that could generate the observed pattern and worth future study.

### Estimation biases

Earlier theoretical work has proven the feasibility of accurately estimating genetic diversity with single individuals [20, 21]. Several studies have used whole-genome sequencing data from a single individual to reconstruct detailed past demographic histories [26, 27]. Application of this individual-based approach allowed us to include species with only handful of museum tissue samples for species comparisons. However, RAD data from non-model organisms present additional challenges including a high rate of sequencing errors and data processing errors (i.e., *de novo* assembly and linkage assignment with a distantly related reference genome). In addition, we multiplexed samples for Illumina sequencing so that RAD loci only covered ~3.3 % of the genome for each individual. Thus, while our study included substantially more data than would have been possible using Sanger sequencing, we nonetheless examined a relatively small fraction of the genome of each species.

To assess the accuracy of our *R*_*Z:A*_ estimates, we extended the maximum likelihood method in Lynch et al. [28] to co-estimate sequencing error rates and heterozygosities. We also found that the *R*_*Z:A*_ estimates are robust to variety of data filtering criterions (Additional file 3: Figure S2). Moreover, we applied multiple steps of quality control to ensure that dichromatic and monochromatic species did not systematically differ regards to either genomic or sequencing coverage of RAD loci (Additional file 3: Figure S2; and see Additional file 6), as the amount of data is not exact equal across individuals because of the shotgun nature of next-generation sequencing and different preservation quality of museum tissues.

One caveat with RAD sequencing that cannot be resolved with increased data quantity is allele dropout — mutations at enzyme cutting sites can result in underestimates of genetic diversity, and this problem is exacerbated as genetic diversity increases [29]. In this study, genetic diversities are low across all individuals (mean heterozygosity is 0.004), suggesting a limited effect of allele dropout. This potential bias is further mitigated by considering the ratio of genetic diversity between Z chromosome and autosomes. More importantly, dichromatic and monochromatic species do not differ significantly regards to genetic diversity (Wilcoxon test *p* > 0.10 for both autosomal and Z-linked diversity). Our use of a distantly related reference genome for linkage assignment is another potential issue leading to underestimated genetic diversity, because more variable RAD markers might be lost during linkage assignment. This problem is not specific to RAD sequencing but would also pertain to Sanger sequencing, which would tend to select for loci that are conserved enough to be amplified across species. However, it would equally affect the *R*_*Z:A*_ estimates of a species pair given that both taxa are equally divergent from the zebra finches. Hence, estimation biases might exist, but is unlikely to contribute to the *R*_*Z:A*_ difference between matching species pairs.

### Factors affecting R_Z:A_

Due to the unique mode of inheritance of sex chromosomes, many processes (e.g., mutation, selection, and recombination) affect sex-linked genetic diversity disproportionally. The default prediction is that the level of genetic diversity on Z or X chromosome should be 75 % of that on autosomes, but it is very difficult to pinpoint the exact cause for deviations in empirical studies because of confounding genetic processes e.g., [30, 31]. Several monochromatic species in our study have *R*_*Z:A*_ estimates lower than the theoretical minimal of 9/16. This ratio arises as the limiting value of the breeding ratio for a polygynous system where a single male has access to a female breeding population of infinite size [18]. Other studies on birds have found even more extreme *R*_*Z:A*_ estimates than those we report here 0.20-0.36; [32–36]. This suggests that the male-to-female breeding ratio *per se* is not the only process that can deplete Z-linked genetic diversity in these species.

Selection is a process that can affect genetic diversity. Because RAD data are drawn from coding and non-coding regions alike, we could not assess the effects of selection (e.g., separating synonymous versus nonsynonymous substitutions). However, our dataset is composed of ~480,000 RAD loci per individual, anonymously selected by restriction enzymes and more or less evenly distributed across the genome (Additional file 2: Figure S1). We thus believe that our data are more likely to reflect genome-wide patterns of variation rather than selection on individual genes. There is possibility that RAD loci’s nucleotide diversity is influenced by background selection and selective sweeps on linked loci [37, 38]. Z chromosomes do not recombine in the heterogametic sex; therefore, the diversity-reducing effect of selection is expected to be stronger. The observed genetic pattern could be due to higher levels of background selection or frequent selective sweeps on the Z chromosome in monochromatic species. The relationship between plumage traits, Z-linked genes and selection is an important and highly relevant area of research. At present, we do not adequately understand the parameter space of selection—how large the selection coefficient has to be, how many genes have to be under selection and how frequent selection has to happen—for chromosome-wide genetic variation to be affected.

Demographic history can differentially affect genetic diversity on the sex chromosomes and autosomes. In Pool and Nielsen [39], coalescent modeling revealed that a historical population bottleneck or expansion alone could produce a wide range of *R*_*Z:A*_ ratios. Specifically, genetic diversity on the Z chromosome drops more dramatically with population size reduction (i.e., lower *R*_*z:A*_), while population size increase leads to a more equal genetic diversity (i.e., higher *R*_*z:A*_). One drawback of estimating *R*_*Z:A*_ from one individual is that the effect of demographic history cannot be quantified without a recombination map. However, we obtained highly correlated *R*_*Z:A*_ estimates among individuals from widely-separated geographic localities (Additional file 1: Table S1). Local population histories might contribute to the differences between individuals; nevertheless, these intra-species differences were relatively insignificant compared to the inter-species differences (Fig. 1). We also tried to minimize the effect of different biogeographic histories by carefully choosing our study species and individual samples. All species selected in this study breed in North America, all species pairs have large overlapping geographic ranges, and museum tissue samples were chosen such that species pairs would have roughly matching geographic sampling (Additional file 1: Table S1). For example, the warbler species pair—Ovenbirds (*Seiurus aurocapilla*) and Black-throated Blue Warbler (*Dendroica caerulescens*)—both have samples from the state of New York and Michigan. There is a possibility that these species pairs chosen specifically for contrasting level of dichromatism somehow were also on contrasting trajectories in terms of past demographic history, and it would be an interesting hypothesis to test for future studies. Related to population demography, population structure is another factor that can influence *R*_*z:A*_. For birds, female-biased dispersal is a common pattern [40], which could reduce *R*_*Z:A*_ as the genetic diversity of the Z chromosome would decrease more due to local inbreeding than that of the autosomes [41]. How dichromatism is correlated with sex-biased dispersal pattern still awaits future study.

### Factors correlated with dichromatism

The intensity of sexual selection is a factor often assumed to be correlated with dichromatism, leading to the widespread use of dichromatism as a surrogate for sexual selection in phylogenetic comparative studies e.g., [10, 42–44]. Our finding that *R*_*z:A*_ is negatively associated with dichromatism seems to be at odds with the current understanding of this trait. Among the eight dichromatic species in this study, the red-and-yellow shoulder badge (“epaulet”) on red-winged blackbirds is considered to have an important role in maintaining male territories, the quality of which is selected by females [45]. The blue-ultraviolet plumage coloration on male eastern bluebirds is a reliable predictor of male reproductive success [7], and female American goldfinches prefer males with brighter bills and plumage in mate-choice experiments [46]. However, many avian studies have found no evidence for strong current sexual selection on sexually dimorphic traits (e.g., red-winged blackbirds [47, 48]; dickcissels [49]). These inconsistencies underline the difficulties in inferring the strength of sexual selection of a species based on phenotypic traits. Some studies have reported geographic variation in the traits under sexual selection [50, 51], and the direction of sexual selection is known to vary through time [52]. Results from these species-specific and trait-oriented studies are often not comparable between species, and hence, do not provide robust evidence for or against the association between dichromatism and sexual selection across species. Nonetheless, a simple decoupling between ongoing sexual selection and dichromatism predicts a lack of correlation between *R*_*z:A*_ and dichromatism level, but cannot explain why we observe a significant negative correlation (Fig. 1). To explain this intriguing pattern, we would need to identify a mechanism that can affect Z-linked genetic diversity and which is also known to be associated with dichromatism. Here, we discuss a few possibilities base on our current knowledge. This list is by no means an exhaustive list and many traits require further investigation.

One possible explanation involves differential mutation rates, which could directly affect genetic diversity (*θ*_*Z*_ = *4N*_*Z*_*μ*_*Z*_). Limited evidence suggests a correlation between plumage dimorphism and testis mass [8]; however, this association is only significant when phylogenetic relationships are ignored: see Table 2 in ref. [8]. If the larger testis mass in dimorphic species is associate with more cell division in males, *R*_*Z:A*_ would become higher in dimorphic species with respect to the monomorphic species because of elevated substitution rates on Z chromosome. A faster-Z effect, in which the Z chromosome has elevated rates of substitution or a higher proportion of nonsynonymous changes, is often detected in studies of bird genome evolution e.g., [53–55]. While the faster-Z effect is also observed among RAD loci (i.e., higher substitution rate on Z chromosome across all species and lineages), we found no significant difference in substitution rates between dichromatic and monochromatic species (Additional file 4: Figure S3). This is concordant with previous studies on mutation rates and sexual selection: an analysis with 32 mammalian genomes did not find significant correlation between sperm competition and male mutation bias [56], and a meta-analysis suggested no correlation between sexual selection and spontaneous mutation rate in birds [57]. A recent analysis with 45 newly sequenced bird genomes also found no significant correlation between fast-Z evolution and a range of life history traits, including dichromatism and tail dimorphism [54]. Admittedly, using ratio of substitution rate (*μ*_*Z*_*/μ*_*A*_) is not a perfect method to correct for male mutation bias, but the pattern we observed is unlikely to be driven by male mutation bias.

Natural selection is another process that has long been proposed to associate with dichromatism in birds [3]. In fact, the hypothesis that natural selection in females drives the evolution of dichromatism has been supported by several recent analyses that examined male and female color evolution separately e.g., [4, 5]. For example, a study in the grackles and allies (Icteridae) found that female plumage color evolves more rapidly than male plumage color, thus suggesting a prominent role for female-mediated natural selection in the evolution of sexual dichromatism [5]. We note that these comparative studies are on a longer time scale than our analysis, because the genetic diversity measurements used here can only reflect changes in recent past (more specifically, the past 4*Ne* generations). Nevertheless, they suggest a possible mechanism: if ongoing natural selection is stronger for females in dichromatic species (e.g., strong selection for being cryptic) resulting in fewer females in the breeding population, the species’ *R*_*Z:A*_ would become higher than monochromatic species. However, this mechanism is not supported by studies on mortality rate, arguably the most salient outcome of natural selection. Studies have revealed that mortality is positively correlated with plumage brightness [58], and that male-biased mortality is correlated with more intense male-male competition [59]. If these findings generalize to our study, dichromatism and male-biased mortality should act in concert to reduce the number of males in the breeding population, which should further reduce *R*_*z:A*_ for dichromatic species. Admittedly, these two studies presented very weak, indirect evidences against present natural selection on female plumage as a possible mechanism, and more studies comparing the strength of natural selection across species using other outcomes (e.g., brood success) would be informative.

Several life history traits have been shown to correlate with plumage dimorphism. The association between social mating system and dichromatism has been examined by many studies e.g., [8, 9], and a recent study in shorebirds found that mating system was significantly correlated with Z-linked genetic diversity [60]. However, the species pairs chosen in our study do not differ appreciably in social mating system (polygyny in Red-Winged Blackbird and Eastern Meadowlark; monogamy in all others). Data on the genetic mating system of birds (e.g., rate of ex-paternity mating) is scant for the monochromatic species in this study. Song complexity is known to be negatively correlated with male carotenoid-based coloration in some groups of passerine birds [61]. It is possible that the monochromatic species we have studied are actually experiencing more intense sexual selection, but targeting this more cryptic trait. This would suggest that song would be a better surrogate for sexual selection at the phenotypic level. Likewise, any other behavior or non-plumage related trait associated with the intensity of sexual selection, but having a trade-off relationship with dichromatism could provide a potential mechanism to explain the lower Z-linked genetic diversity in monochromatic species.

## Conclusions

Here, we developed a RAD-based approach to estimate neutral genetic diversity on sex chromosomes in relation to that on autosomes (*R*_*Z:A*_) for non-model avian species. We used this framework to test the association between sex-linked genomic variation and avian plumage dichromatism, a widely used surrogate for sexual selection. We documented an intriguing pattern of reduced *R*_*Z:A*_ ratios in monochromatic species relative to phylogenetically-matched dichromatic species. If the patterns reported here are caused by reduced or even similar levels of sexual selection in dichromatic species, and if these results are generalizable to other avian taxa, then our results have broad implications for the comparative study of speciation rates in relation to sexual selection in birds. We are presently unable to explain this pattern, but we predict that the solution to the paradox lies in understanding the potentially complex tradeoffs between dichromatism and a host of ecological or life-history traits [62]. The multiple interpretations of our findings suggest limits to our understanding of the association between sexual selection, plumage dichromatism and genetic diversity.

## Methods

### Study species pairs and tissue samples

We used bird tissue samples from the collections in UMMZ (University of Michigan-Museum of Zoology), CUMV (Cornell University-Museum of Vertebrates) and MVZ (The Museum of Vertebrate Zoology at Berkeley). Our RAD-based approach estimates an *R*_*Z:A*_ for each individual bird. This minimal sampling requirement allowed us to include bird species with limited number of tissue samples in the study. We selected tissue samples of the homogametic sex (i.e., male) according to museum records so that individual estimates of Z-chromosome genetic diversity could be obtained. As the first application of our approach, we focused on species with multiple samples from widely-separated geographic locations so that we can assess both the intra- and inter-species differences of *R*_*Z:A*_ estimates. Eight pairs of species from seven different families (Additional file 1: Table S1) were included in this study. As our approach involves mapping RAD sequences to a reference genome for linkage identification, all chosen species are passerine birds except two species pairs from the family of woodpeckers (Picidae). The selected monochromatic species all have indistinguishable plumage between sexes, but there are both monochromatically cryptic species (e.g., Ovenbird) and monochromatically conspicuous species (e.g., Red-headed Woodpecker). The dichromatic species show a range of dichromatic levels – ranging from dramatic intersexual plumage differences (e.g., the sexes were once identified as separate species for Black-throated Blue Warbler and William’s Sapsucker) to more subtle differences (e.g., Red-breasted Nuthatch); yet, in all cases, male is the more conspicuous and/or colorful sex, which fits the prediction of Darwinian sexual selection.

### Next-generation Sequencing

Genomic DNA of 41 selected tissue samples (2-4 individuals per species) was first used to confirm that the individual was the homogametic sex using DNA-based sex identification methods [63, 64], and then digested with *PstI* and *MseI* restriction enzymes. Digested fragments were barcoded and size-selected (150-250 bp) to generate a multiplexed Double Digested RADseq library [65]. Samples were split for sequencing—five species pairs (24 samples) were sequenced (100 bp paired-end) on one lane of Illumina HiSeq2000 sequencer at University of Michigan DNA Sequencing Core, while the other three pairs (18 samples) were multiplexed with unrelated samples and sequenced on Illumina HiSeq2500 at the Next-Generation Sequencing Facility at the Hospital for Sick Children in Toronto.

### Data processing

After illumina sequencing, the raw data was de-multiplexed into individual datasets according to the barcodes. Analyses were the performed on each individual dataset separately, unless otherwise noted. Here, we briefly explain the basic approach, while the detailed steps, parameter settings and statistics are reported in the Additional file 6 (scripts for data processing can be found on Dryad Digital Repository: 10.5061/dryad.55044).

As there is no closely related species with assembled genome sequence for our selected species, *de novo* assembly methods were used to identify putative RAD loci. The consensus sequences of *de novo* RAD loci were blasted against the Zebra Finch genome WUSTL v3.2.4 assembly [66]; to determine their genomic origins (i.e., Z-associated or autosomal loci). The Z-associated and autosomal genetic diversity (*θ*_*Z*_ and *θ*_*A*;_ where *θ = 4Nμ*; *N* is the effective population size and *μ* is the substitution rate) were co-estimated with the sequencing error rate using an extension of the maximum likelihood (ML) method in [28]. Briefly, in the original ML framework [28], *θ* and the sequencing error rate were jointly estimated across all loci; here we put in two parameters for *θ— θ*_*Z*_ and *θ*_*A*_ – according to the genomic locations of RAD loci, and jointly estimated the three parameters. We further applied several data filtering criterions to assess the robustness the estimates of the genetic diversity ratio (*θ*_*Z*_/*θ*_*A*_) are to assembling and mapping errors (Additional file 3: Figure S2; see Additional file 6 for details).

Using the zebra finch reference genome, we calculated the substitution-rate ratio (*μ*_*Z*_/*μ*_*A*_) for the RAD loci in two ways. The first approach is based on individual datasets. We calculated the substitution rates from the percentage of fixed differences between individual’s RAD loci and the reference genome. These individual estimates of substitution-rate ratio were used to correct the individual *R*_*Z:A*_ estimates (Fig. 1 and Additional file 3: Figure S2) for male-mutation bias. The second approach pools data across individuals of the same species pair. For the genomic regions that were mapped by both dichromatic and monochromatic species, we could assign the difference between RAD loci and the reference genome to lineages (Additional file 4: Figure S3), and calculate lineage-specific substitution rates for the dichromatic and monochromatic species. Hence, we obtain an estimate of substitution-rate ratio for each species using combined data across individuals. The divergent time used to calculate absolute substitution rates was extracted from a recently published time-calibrated maximum clade credibility (MCC) phylogeny for all birds [67].

### Statistical analyses

Three different statistical analyses were used to test the differences between dichromatic and monochromatic species’ estimates of *R*_*Z:A*_. Wilcoxon signed-rank test was used to testing the differences between species means (i.e., mean *R*_*Z:A*_ across sampled individuals). Because we chose matched pairs of dichromatic and monochromatic species (Fig. 1 and Additional file 1: Table S1), the contrasts between species pairs are independent.

Linear regression models with mixed effect were used for individual *R*_*Z:A*_ estimates:

$$ {R}_{Z:A}\sim {\beta}_0+{\beta}_p+{\beta}_dd+{u}_s $$

This model estimates the effect of dichromatism (*β*_*d*_, *d* as a indicator variable for dichromatism) while allowing different baseline *R*_*Z:A*_ for different species pairs (*β*_*p*_), and accounting for the fact that individual estimates of the same species are not independent (${u}_s$, random intercept for each species). Comparing this model to a reduced model (*β*_*d*_ = 0; no effect of dichromatism) using likelihood ratio test could assess whether the effect of dichromatism is significant (i.e., whether *β*_*d*_ is significantly differ from zero). This mix-effect model was also used for testing whether species differ in substitution rate (μ) and ratio (μ_z_/μ_A_).

Lastly, we also used phylogenetic generalized least squares (PGLS) analysis [68] to explicitly controlled for phylogeny. The phylogeny of our studied species were extracted from the MCC tree in [67]. Multiple samples per species were added as additional tips to the phylogeny. For computation, we set the lengths of these tip branches to a small value (i.e., 0.01 Myr; varying the length from 0.001 to 0.05 did not affect the significant level of the *p* values). We estimated the amount of phylogenetic signal using Pagel’s λ [69], while fitting the PGLS models using the caper package [70] in R [71]. This PGLS analysis was also used for quantitative measurements of dichromatism (Fig. 2)—14 species in our study has reflectance-based measurements from spectrophotometer published in [24].

## Availability of supporting data

Additional Methods could be found in the Additional file 6, and the data and analysis scripts are archived and available for download on Dryad (DOI: 10.5061/dryad.55044).

## Additional files

Additional file 1: Table S1. Matching species pairs and the geographic locations (U.S. states) of sampled birds. (DOCX 88 kb) Additional file 2: Figure S1. Genomic data summary. (A) Proportion of total sequence data (144 million paired-end sequences) obtained for each of the forty-one samples—2-4 individuals from distinct geographic locations per species. Light and dark paired colors represent matching dichromatic and monochromatic species pairs. (B) Number of loci identified by *de novo* assembly for each sample. (C) Genomic distribution of RAD loci shown by the number of base pairs mapped by RAD sequences for each million base pair window on the Zebra Finch genome. Sister species have similar genomic distribution profiles—plot shows individuals from the species pair of Eastern Bluebird (plotted outward) and Veery (inward). Chromosomes are in different colors and grey indicates uncertainty in the reference genome assembly (e.g., ChrUN is a collection of contigs that could not be confidently assigned to chromosomes). (D) Sequencing coverage and genomic coverage (i.e., the total alignment length between mapped RAD loci and the reference genome) of each sample’s mapped and filtered dataset used for estimating genetic diversities and mutation rates on the Z chromosome and autosomes. Coverage varies across individuals (averages: vertical dashed lines); in particular, variation in genomic coverage suggests the effect of phylogenetic distance to the reference genome – more distantly related species have fewer RAD loci mapped. Yet, no consistent differences were noted between dichromatic and monochromatic species (*p* values from linear regression controlling for the effect of sequencing runs and phylogenetic distance to the reference genome were 0.12 and 0.67, respectively). (PDF 577 kb) Additional file 3: Figure S2. Estimates of genetic diversity ratio (${\theta}_Z/{\theta}_A$; A) and *R*_*Z:A*_ (i.e., corrected for mutation-rate biases; B) across different criterions of data filtering for matched pairs of sexually dichromatic (red) and monochromatic (black) bird species. Lines connected estimates from the same sampled individual. Species pairs are arranged such that the phylogenetic distance to Zebra Finch increases from left to right. Seven different filtering criterions were used. In the first round, all mapped RAD loci with coverage higher than one and lower than the individual cutoff (i.e., mean coverage plus two times standard deviation) were used, and the second round excluded RAD loci with either more than 5 % variable sites, or more than 4 variable sites segregated in 10bp fragment, or more than 20 % sequence divergence from the reference genome. In additional to the second round filtering, we applied another six filters: only including genomic regions shared between the monochromatic and dichromatic species in species pairs; only using mapped RAD loci with sequence coverage ≥5; only using mapped RAD loci with ≤5 %, ≤10 %, ≤15 % sequence divergence from zebra finch; excluding loci from micro-chromosomes (i.e., only have autosomal loci from Chromosome 1-10). For plotting, estimates higher than 1.5 were not shown (marked as x; multiple occurrences for woodpeckers and sapsuckers with stringent divergence filters). Dichromatic species consistently have higher estimates compared to their paring monochromatic species- the highest *p* values from Wilcoxon signed-rank test on species means were 0.04 (${\theta}_Z/{\theta}_A$) and 0.02 (*R*_*Z:A*_) with the first round estimates. (PDF 481 kb) Additional file 4: Figure S3. Substitution-rate ratios for matched pairs of sexually dichromatic (red) and monochromatic (black) bird species. Diagram in top panel illustrates the two types of estimates plotted: individual estimates (filled dots, means of species pairs connected by solid lines) and lineage estimates (open dots, species pairs connected by dash lines). The former was calculated by directly comparing each individual’s mapped RAD loci to the zebra finch genome. Pooling mapped RAD loci across individuals of species pairs, and counting the number of mutations specific to the dichromatic- or monochromatic- species lineage obtained the later, so only one value per species. Individual estimates are lower in dichromatic species (*p* <0.001 from mixed-effect linear regression), while lineage estimates do not significantly differ regards to dichromatism (*p*=0.84 from Wilcoxon test). (PDF 399 kb) Additional file 5: Figure S4. Substitution rates of autosomal loci (A and C; y-axis unit is 10^-9^ per site per year) and ratios of substitution rates (B and D) for matched pairs of sexually dichromatic (red) and monochromatic (black) bird species under different divergence cutoffs. Besides each plot, *p* values from testing the difference between dichromatic and monochromatic samples are reported. Mixed-effect linear regressions were used for individual estimates (A and B), while Wilcoxon signed-rank tests were applied to lineage estimates (C and D). (PDF 468 kb) Additional file 6: Supplementary Methods. Detailed steps of the data analysis, related quality controls and filtering criterions and summary statistics of RAD data. (DOCX 1496 kb)
