# Supplementary material for: Sex-linked genomic variation and its relationship to avian plumage dichromatism and sexual selection
Source: BMC Evol Biol. 2015 Sep 16;15:199. doi: 10.1186/s12862-015-0480-4 (PMC4574164; doi:10.1186/s12862-015-0480-4)
Supplement: Additional file 1: Table S1. — Matching species pairs and the geographic locations (U.S. states) of sampled birds. (DOCX 88 kb) [file 12862_2015_480_MOESM1_ESM.docx]

**Table S1** Matching species pairs and the geographic locations (U.S. states) of sampled birds.

| **Family** | **Monochromatic species** | **Dichromatic species** |
| --- | --- | --- |
| **Parulidae (Warblers)** | **Ovenbird**  *Seiurus aurocapilla* | **Black-throated Blue Warbler**  *Dendroica caerulescens* |
|  | New York | New York |
|  | Michigan | Michigan |
|  |  | North Carolina |
| **Icteridae (Blackbirds)** | **Eastern/Western Meadowlark**  *Sturnella magna/neglecta* | **Red-winged Blackbird**  *Agelaius phoeniceus* |
|  | Louisiana | Texas |
|  | California | Oregon |
|  | Michigan |  |
| **Fringillidae (Finches)** | **Pine Siskin**  *Carduelis pinus* | **American Goldfinch**  *Carduelis tristis* |
|  | Michigan | Michigan |
|  | New York | New York |
|  |  | Colorado |
| **Turdidae (Thrushes)** | **Veery**  *Catharus fuscescens* | **Eastern Bluebird**  *Sialia sialis* |
|  | Michigan | Michigan |
|  | New York | New York |
| **Sittidae (Nuthatches)** | **Pygmy Nuthatch**  *Sitta pygmaea* | **Red-breasted Nuthatch**  *Sitta canadensis* |
|  | California (x2) | California |
|  | Nevada | Michigan |
|  |  | New York |
| **Hirundinidae (Swallows)** | **Northern Rough-winged Swallow**  *Stelgidopteryx serripennis* | **Purple Martin**  *Progne subis* |
|  | Michigan | Michigan |
|  | New York | North Carolina |
| **Picidae (Woodpeckers and Sapsuckers)** | **Red-headed Woodpecker**  *Melanerpes erythrocephalus* | **Red-bellied Woodpecker**  *Melanerpes carolinus* |
|  | Alabama | Florida |
|  | Texas | Texas |
|  | Michigan | Michigan |
|  | **Red-breasted Sapsucker**  *Sphyrapicus ruber* | **Williamson's Sapsucker**  *Sphyrapicus thyroideus* |
|  | California | California (x2) |
|  | Washington | Oregon |
|  |  | Montana |
