# Supplementary material for: Sex-linked genomic variation and its relationship to avian plumage dichromatism and sexual selection
Source: BMC Evol Biol. 2015 Sep 16;15:199. doi: 10.1186/s12862-015-0480-4 (PMC4574164; doi:10.1186/s12862-015-0480-4)
Supplement: Additional file 2: Figure S1. — Genomic data summary. (A) Proportion of total sequence data (144 million paired-end sequences) obtained for each of the forty-one samples—2-4 individuals from distinct geographic locations per species. Light and dark paired colors represent matching dichromatic and monochromatic species pairs. (B) Number of loci identified by de novo assembly for each sample. (C) Genomic distribution of RAD loci shown by the number of base pairs mapped by RAD sequences for each million base pair window on the Zebra Finch genome. Sister species have similar genomic distribution profiles—plot shows individuals from the species pair of Eastern Bluebird (plotted outward) and Veery (inward). Chromosomes are in different colors and grey indicates uncertainty in the reference genome assembly (e.g., ChrUN is a collection of contigs that could not be confidently assigned to chromosomes). (D) Sequencing coverage and genomic coverage (i.e., the total alignment length between mapped RAD loci and the reference genome) of each sample’s mapped and filtered dataset used for estimating genetic diversities and mutation rates on the Z chromosome and autosomes. Coverage varies across individuals (averages: vertical dashed lines); in particular, variation in genomic coverage suggests the effect of phylogenetic distance to the reference genome – more distantly related species have fewer RAD loci mapped. Yet, no consistent differences were noted between dichromatic and monochromatic species (p values from linear regression controlling for the effect of sequencing runs and phylogenetic distance to the reference genome were 0.12 and 0.67, respectively). (PDF 577 kb) [file 12862_2015_480_MOESM2_ESM.pdf]

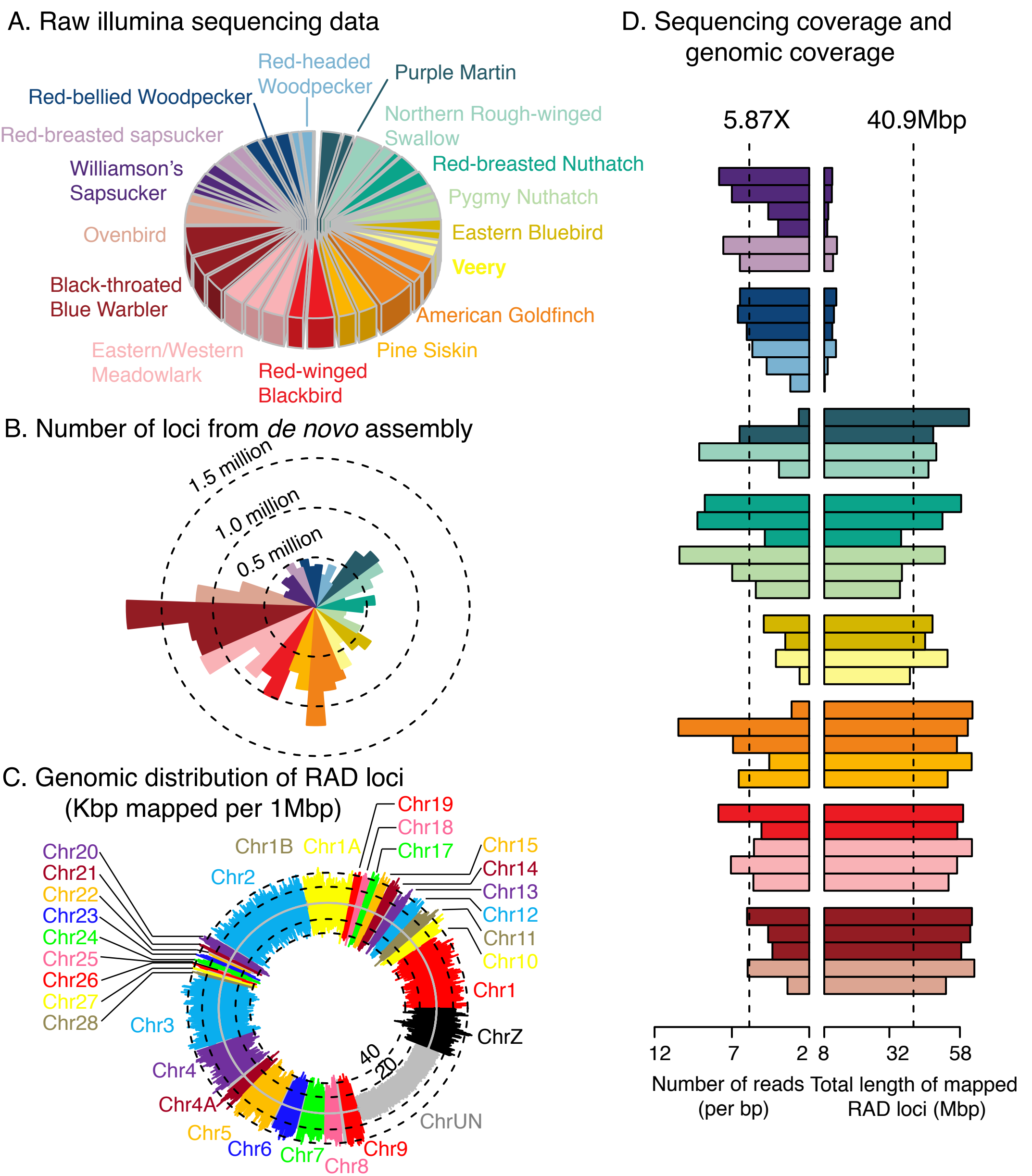

A. Raw illumina sequencing data
Red-headed Woodpecker
Purple Martin
Red-bellied Woodpecker
Northern Rough-winged Swallow
Red-breasted sapsucker
Red-breasted Nuthatch
Williamson's Sapsucker
Pygmy Nuthatch
Ovenbird
Eastern Bluebird
Veery
Black-throated Blue Warbler
American Goldfinch
Eastern/Western Meadowlark
Pine Siskin
Red-winged Blackbird
B. Number of loci from de novo assembly
1.5 million
1.0 million
0.5 million
C. Genomic distribution of RAD loci (Kbp mapped per 1Mbp)
Chr19
Chr18
Chr1B
Chr1A
Chr17
Chr15
Chr14
Chr13
Chr12
Chr11
Chr10
Chr20
Chr21
Chr22
Chr23
Chr24
Chr25
Chr26
Chr27
Chr28
Chr2
Chr1
ChrZ
Chr3
Chr4
Chr4A
Chr5
Chr6
Chr7
Chr8
Chr9
ChrUN
40
20
D. Sequencing coverage and genomic coverage
5.87X
40.9Mbp
12
7
2
8
32
58
Number of reads (per bp)
Total length of mapped RAD loci (Mbp)
