# Supplementary figures and images for: Sex-linked genomic variation and its relationship to avian plumage dichromatism and sexual selection
Source: BMC Evol Biol. 2015 Sep 16;15:199. doi: 10.1186/s12862-015-0480-4 (PMC4574164; doi:10.1186/s12862-015-0480-4)

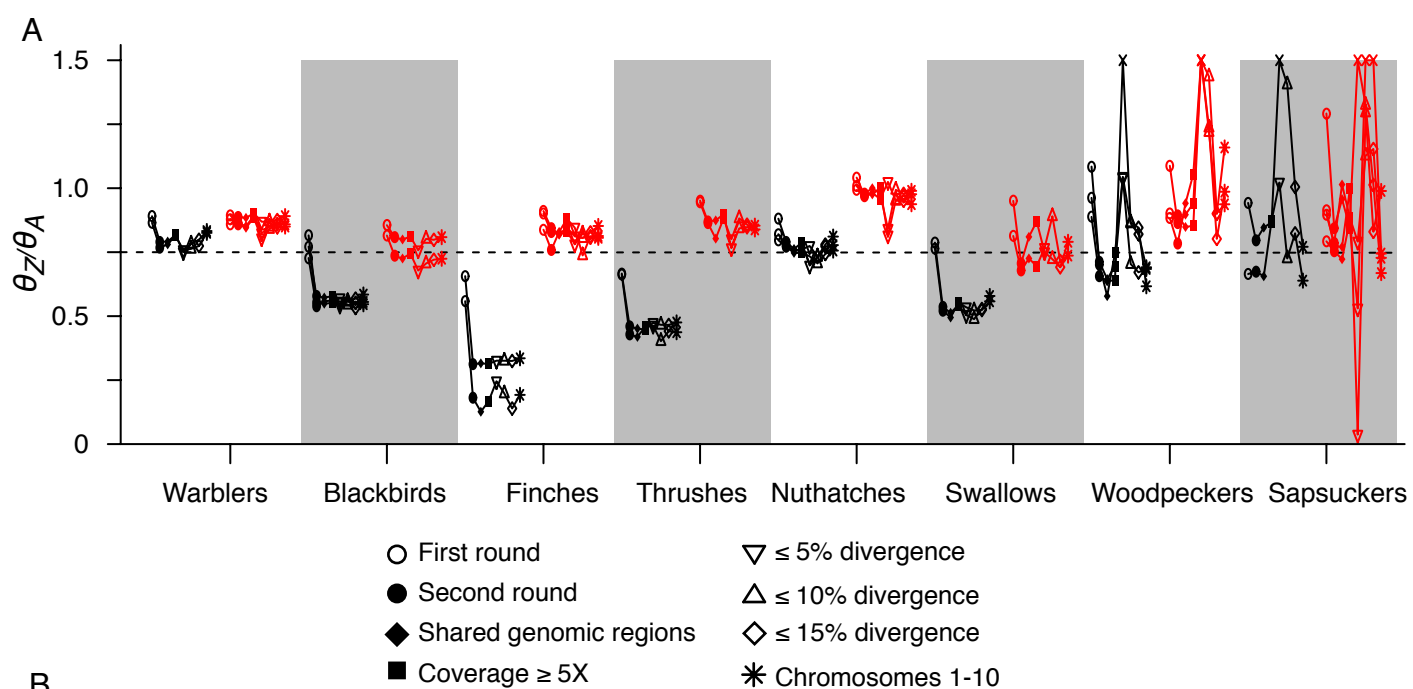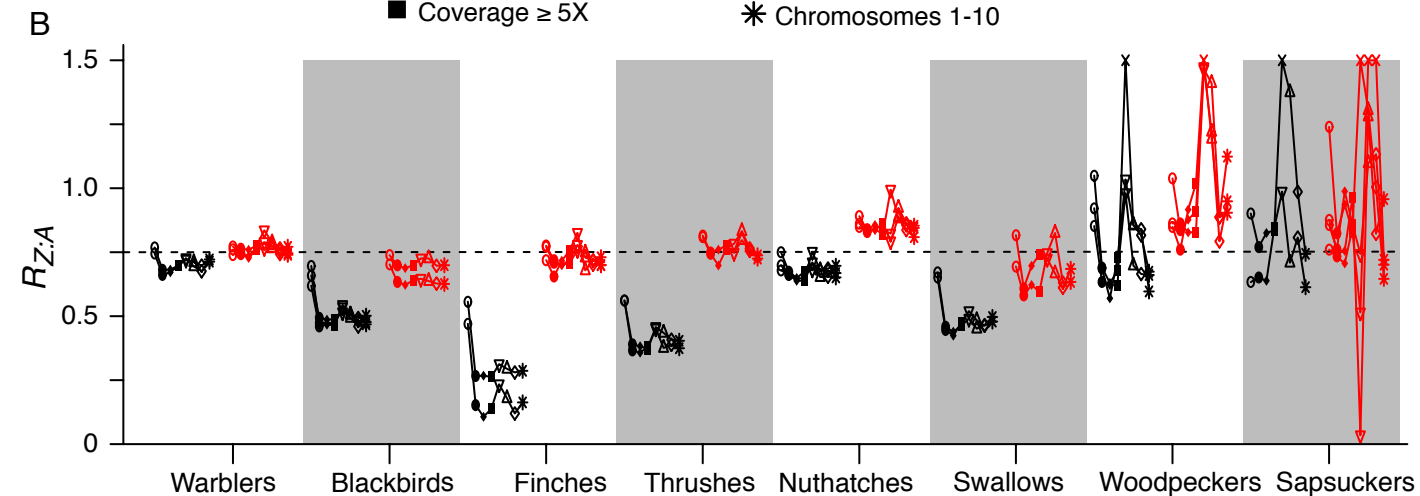

Supplement: Additional file 3: Figure S2. — Estimates of genetic diversity ratio (${\theta}_Z/{\theta}_A$θZ/θA; A) and R Z:A (i.e., corrected for mutation-rate biases; B) across different criterions of data filtering for matched pairs of sexually dichromatic (red) and monochromatic (black) bird species. Lines connected estimates from the same sampled individual. Species pairs are arranged such that the phylogenetic distance to Zebra Finch increases from left to right. Seven different filtering criterions were used. In the first round, all mapped RAD loci with coverage higher than one and lower than the individual cutoff (i.e., mean coverage plus two times standard deviation) were used, and the second round excluded RAD loci with either more than 5 % variable sites, or more than 4 variable sites segregated in 10bp fragment, or more than 20 % sequence divergence from the reference genome. In additional to the second round filtering, we applied another six filters: only including genomic regions shared between the monochromatic and dichromatic species in species pairs; only using mapped RAD loci with sequence coverage ≥5; only using mapped RAD loci with ≤5 %, ≤10 %, ≤15 % sequence divergence from zebra finch; excluding loci from micro-chromosomes (i.e., only have autosomal loci from Chromosome 1-10). For plotting, estimates higher than 1.5 were not shown (marked as x; multiple occurrences for woodpeckers and sapsuckers with stringent divergence filters). Dichromatic species consistently have higher estimates compared to their paring monochromatic species- the highest p values from Wilcoxon signed-rank test on species means were 0.04 ( [file 12862_2015_480_MOESM3_ESM.pdf]

Individual  
estimates

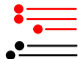

Zebra Finch  
Genome

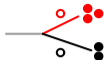

Lineage  
estimates

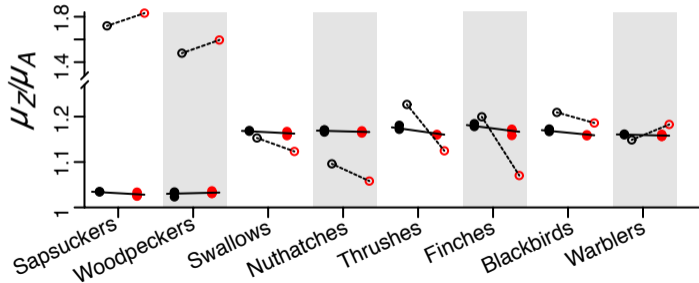

Supplement: Additional file 4: Figure S3. — Substitution-rate ratios for matched pairs of sexually dichromatic (red) and monochromatic (black) bird species. Diagram in top panel illustrates the two types of estimates plotted: individual estimates (filled dots, means of species pairs connected by solid lines) and lineage estimates (open dots, species pairs connected by dash lines). The former was calculated by directly comparing each individual’s mapped RAD loci to the zebra finch genome. Pooling mapped RAD loci across individuals of species pairs, and counting the number of mutations specific to the dichromatic- or monochromatic- species lineage obtained the later, so only one value per species. Individual estimates are lower in dichromatic species (p <0.001 from mixed-effect linear regression), while lineage estimates do not significantly differ regards to dichromatism (p=0.84 from Wilcoxon test). (PDF 399 kb) [file 12862_2015_480_MOESM4_ESM.pdf]

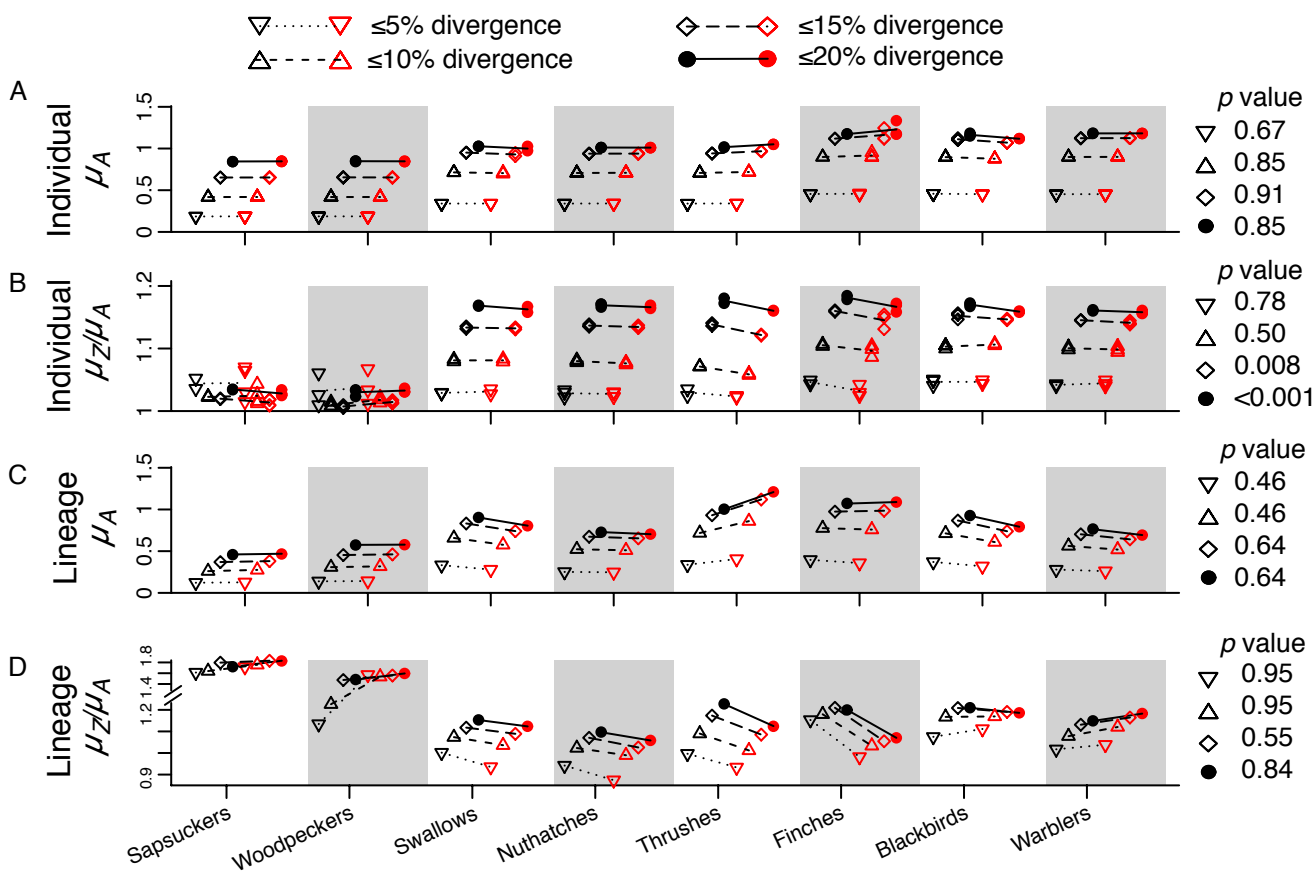

Supplement: Additional file 5: Figure S4. — Substitution rates of autosomal loci (A and C; y-axis unit is 10-9 per site per year) and ratios of substitution rates (B and D) for matched pairs of sexually dichromatic (red) and monochromatic (black) bird species under different divergence cutoffs. Besides each plot, p values from testing the difference between dichromatic and monochromatic samples are reported. Mixed-effect linear regressions were used for individual estimates (A and B), while Wilcoxon signed-rank tests were applied to lineage estimates (C and D). (PDF 468 kb) [file 12862_2015_480_MOESM5_ESM.pdf]
