# Supplementary material for: Sex-linked genomic variation and its relationship to avian plumage dichromatism and sexual selection
Source: BMC Evol Biol. 2015 Sep 16;15:199. doi: 10.1186/s12862-015-0480-4 (PMC4574164; doi:10.1186/s12862-015-0480-4)
Supplement: Additional file 6: Supplementary Methods. — Detailed steps of the data analysis, related quality controls and filtering criterions and summary statistics of RAD data. (DOCX 1496 kb) [file 12862_2015_480_MOESM6_ESM.docx]

**Supplementary Methods:**

*Sequencing Data Processing*

Starting from the raw Illumina sequencing data, the data analysis included four steps: 1) demultiplexing data to prepare separated sequence files for each individual; 2) identifying putative RAD loci by *de novo* assembly; 3) separating autosome loci from Z-linked loci by mapping RAD loci to a reference genome; 4) estimating *θ* for the Z-linked and autosomal RAD loci; and 5) estimating the substitution rates for mapped RAD loci. Customized Python, Perl and R scripts used in the analysis were deposited to Dryad along with the data (XXX) and following sections will describe each step and reported related summary statistics in details. All steps after demultiplexing were performed on each individual’s data file separately, and the statistics reported are averages and standard deviations across individuals, unless noted otherwise.

*1. Preparing individual sequence files for analysis—*Data was de-multiplexed to each individual by identifying and sorting the barcodes with a customized Python script, which also trimmed off the barcode and restriction sites from the sequences. Maximum two sequencing errors were allowed in the barcode plus enzyme recognition sequence and reads with more than one uncalled nucleotide (i.e., ‘N’) were discarded. We excluded the individual with lowest number of reads because following analysis suggested that the amount of data is insufficient to provide converged estimate of heterozygosity (see below). The average number of read pairs for the remaining 41 individuals is 3,513,292 (±1,710,454). Because the two separated sequencing runs provided different amount of raw data (4.2 vs. 2.7 million reads per individual), we used two-way ANOVA to test whether the dataset size differ between dichromatic versus monochromatic samples, and found no significant difference (two-way ANOVA; *p*=0.28).

We further filtered the data using the *fastq_filter* command in USEARCH [1]. Reads were truncated from the first base pair with Q score equal or lower than three, and reads shorter than 20bp after truncation were excluded. We also calculated the expected number of errors from the quality scores, and discarded the reads with error higher than one.

*2. De novo assembly to identify putative RAD loci—* Because there is no closely related species with assembled genome sequence for any of our selected species, we performed *de novo* assembly for identifying putative RAD loci. To speed up the process, the reads were first de-replicated using the *derep-prefix* command, and then clustered according to similarity (95% as identity cutoff) using the *uclust* function in USEARCH [1]. Paired reads were clustered separately for reducing the computation time, but were considered for loci assignment (Fig. S5). That is, a putative RAD locus is a set of read pairs that were grouped into one cluster for both the forward and reverse reads. Individual reads were pooled according to loci assignment and re-aligned using software MUSCLE (Edgar 2004), and the majority consensus sequence for each RAD locus was extracted for next step (Fig. S5). We ignored possible overlaps between read pairs in analysis so far for several reasons. Our size selection range suggests that only a small proportion of the reads would overlap, and the overlap segments are likely to be short. Given that reads usually drop in sequencing quality towards the end (i.e., where the overlap segments locate), merging read pairs just by the sequences themselves would be error-prone. Moreover, the next step is to map the RAD loci to a reference genome, and the mapping result itself could reveal whether a read pair overlaps or not (i.e., whether they are mapped to the same genomic location). On average, individuals have 665,614 (±341,773) RAD loci, sequenced at 5.22 (±1.68) coverage. The number of RAD loci for each individual is positively correlated with the number of reads (linear regression, *p<*0.001), but neither the loci number nor coverage shows difference between dichromatic versus monochromatic species (two-way ANOVA; *p*=0.15 and 0.96, respectively).

*3. Mapping RAD loci to chromosomes*—the chromosomal origin of RAD loci were determined by blasting their consensus sequences against the zebra finch (*Taeniopygia guttata*) genome. A local BLAST database was built from the latest zebra finch genome assembly (WashU taeGut324/taeGut2 assembly; [2]) downloaded from the UCSC Genome Browser database (http://genome.ucsc.edu/). *Windowmasker* was used to mask the over-represented and low complexity sequences on the genome (Morgulis et al. 2006) before building the database with the *makeblastdb* program in the BLAST+ package [3]. Considering the divergent time between zebra finch and our sampled species (33-46 Mya), we started with a set of less stringent parameters (75% conserved sequences) for blast searching with the *blastn* program [4]. The two sides of RAD loci were blasted separately as the intervals between these read pairs were unknown. As expected given the low similarity requirement, majority of the RAD loci have at least one hit (>99%).

The drawback of using less stringent setting is that many blast hits are redundant and non-specific—one RAD locus could be mapped to multiple genomic regions and vice versa. Yet, an ideal mapping for determining the genomic location of RAD loci would be a one-to-one relationship between RAD loci and zebra finch genomic regions. Hence, we filtered the blasting results in two steps (Fig. S6). First, filtering out extra hits for each RAD locus. Since each RAD locus is composed of a pair of sequences, the relative mapping position of paired sequences was examined— the combination of blast hits that has the minimal range on the zebra finch genome as well as the minimal *evalue* (calculated as the product of pairs’ *evalues*) was selected (Fig. S6A). If only one of the pair had blast hit, the locus were noted as “single-mapping” locus, and if there is no combination of blast hits that resided within 50kb on the reference genome, top hits were chosen for each section separately (i.e., minimal *evalue* with at least 40bp sequence alignment) and the locus were noted as “conflicting” locus (Fig. S6B). We chose to keep these single and conflicting mapping for now because they are not necessarily errors; possible biological and experimental causes are: i) high sequence divergence; ii) genomic structure difference between zebra finch and interested species (e.g., genome rearrangement, large insertions and deletions [5]); iii) incomplete assembly of the zebra finch genome (e.g., it has a 174Mbp long “ChrUN” containing sequences that could not be confidently placed to a chromosome); iv) chimeric sequences that can form during PCR in the library preparation [6]. Few RAD loci were filtered out in this step (<1%). Among the remaining loci, a small proportion is single-mapping (<1%), and 44% (±15%) were conflicting loci.

The second step filtered extra hits for each genomic region (see Fig. S6C for illustration). If a genomic region was mapped by multiple RAD loci, the one with longest alignment is more likely to be a true orthologous locus. We calculated the number of un-aligned basepairs (Fig. S6C) for each RAD locus, and deleted the ones with more than 10bp of un-aligned sequences. Some characteristics of the genomic sequence might also cause the multiple blast hits. For example, low-complexity sequences could lead to artificial blasting hits [7] and regions containing gene families could be aligned to multiple paralogous loci. For these regions, it would be difficult in distinguish “true” orthologous hits from erroneous hits. Therefore, we chose to delete the genomic regions with too many hits. For each genomic region, the number of aligned clusters (see Fig. S5), which should represent considerably distinct sequences (<95% similarity), was calculated. Within-individual polymorphisms are very unlikely to generate haplotypes belonging to two clusters, except for those at enzyme cutting sites— clustering algorithm might failed to recover some RAD loci with different starting positions (see Fig. S6D). Hence, genomic regions with more than two consensus clusters were filtered (Fig. S4D). This second step filtered 17.8% (±11.6%) loci, leaving 508,074(±172,674) loci per individual, and changed some of the conflicting mappings to single-mapping locus (among the remaining loci, 28.1±10.2% were singles and 18.1 ±12.3% were conflicting locus).

After the two filtering steps, there were still a small proportion of RAD loci aligned to multiple regions (i.e., with equivalent *evalues*) in addition to regions mapped by two clusters. We utilized the *uclust* function again to exclude potential incorrect mappings (Fig. S6E). Inclusive sets of genomic regions and RAD loci were identified (Fig. S6E), and for each set, the sequences of zebra finch genomic regions and the reads of RAD loci were pooled together to run *uclust* with 75% identity cutoff [1]. For simplicity, RAD pairs were considered separately for identifying inclusive sets. Resulting clusters contained no zebra finch genomic sequences were considered as possible errors in blasting search. Clusters with more than one genomic sequences were considered as potential paralogous mapping and deleted, except when the genomic sequences were identical, which might be due to genome assembly error or recent duplications specific to the zebra finch lineage.

There are on average 484,439 (±231,117) remaining clusters, hereafter referred as mapped RAD loci. The number of mapped RAD loci for an individual would be affected not only by the amount of raw sequencing data to start with, but also by the species’ phylogenetic distance to zebra finch—more distantly related species would have fewer sequences aligned. Hence, we coded the phylogenetic distances as a factor (four different distances between study species and zebrafinch, see Fig. 1 and Table S1), and used it together with the factor of sequencing lane and dichromatism in a multivariate linear regression model to test whether dichromatic and monochromatic samples differ in terms of the dataset size. We found no difference in the number of mapped RAD loci regards to dichromatism level (*p=*0.14). *P* values reported hereinafter were all obtained from this linear regression model unless noted otherwise.

*4. Estimating* *θ* —next-generation sequencing is known for having high sequencing errors compared to traditional Sanger sequencing [8], so a direct count of segregating sites among reads would hugely overestimate the genetic diversity. We adopted a modified maximum-likelihood (ML) framework from Lynch [9] to jointly estimate the sequencing error rate ($\varepsilon$) and heterozygosity (*H;* the probability that a site is a heterozygotes). Briefly, the likelihood function in Lynch [9] considers each site’ likelihood as the sum of two probabilities—being a homozygous or heterozygous site:

$$P_{Homo}=\left( 1-H \right)\cdot\sum_{i=A, G, T,C} p_{i}\cdot b(n-n_{i};n,\varepsilon)$$

$$P_{Hete}=H\cdot\sum_{i=A, G, T,C} \sum_{j\neq i} 2p_{i}p_{j}\cdot b\left( n-n_{i}-n_{j};n,\frac{2\varepsilon}{3} \right)\cdot p({n_{i}; n}_{i}+n_{j}, 0.5)/(1-\sum_{i=A,G,T,C} p_{i}^{2})$$

where *n* is a integer referring to the number of times a site has been sequenced, *n_i_* (*i= A, G, C or T*) describes the sequence profile—the number of times each nucleotide presents among reads, *p_i_*  is the nucleotide frequency, and the two probability functions— $b(n-n_{i};n,\varepsilon)$ and $p({n_{i}; n}_{i}+n_{j}, 0.5)$-- represent the binomial probability of errors (i.e., having $n-n_{i}$ of reads with errors out of *n* reads) and the probability that one allele at the a heterozygous site is sequenced $n_{i}$ times out of $n_{i}+n_{j}$ times, respectively.

In Lynch [9], the product of likelihood across all the sites was maximized, which gives one estimate of heterozygosity (*H*) for the whole data set. Here, the genomic locations of the loci were known from blasting, so we extended this ML framework to incorporate different heterozygosity parameters for different chromosomes. Specifically, we categorized loci into three groups: i) loci unambiguously aligned to Z chromosome sequences with heterozygosity noted as *H_z_*; ii) loci only aligned to mitochondrial sequences with heterozygosity set to zero (i.e., all the nucleotide variations observed at these loci should be due to sequencing errors) and iii) loci unambiguously aligned to autosome sequences with heterozygosity noted as *H_A_*, while the error rate ($\varepsilon$) remained as one parameter shared across all loci. That is, the heterozygosities of Z chromosome and autosomes were co-estimated with the sequencing error rate.

We ignored loci mapped to identical sequences from the Z chromosome and autosomes because of the linkage uncertainty, and also further filtered loci according to the sequencing coverage—loci with only one read do not contain any information about within-individual polymorphism, and loci with too many reads (i.e., more than two standard deviations above the average; same criterion used in *Stacks* [10]) are potential paralogous assembly. A read pair was counted as one read if they present in the same mapped RAD loci. As mapped RAD loci could differ in length, the dataset size will be reported in base pairs instead of loci number below. On average, this initial round of parameter estimation is based on 45,867,526bp (±22,037,193) mapped RAD loci, which were sequenced at 6.44 (±2.12) coverage. 5.66% (±0.65%) of the sequences were Z-linked, and they had almost equal sequencing coverage (coverage ratio between Z-linked and autosomal loci was 0.98±0.03). Differences between dichromatic and monochromatic samples were not significant regards to total sequence length (multivariate linear regression; *p*=0.12), and overall sequencing coverage (*p*=0.67). The proportion of Z-linked sequences is slightly higher in dichromatic species (0.15% more Z-linked sequences in dichromatic species; *p*=0.02) but no difference in relative sequencing coverage (*p*=0.79). Four independent optimization searches with different initial values were conducted using the *optim* function in R [11] to confirm the convergence, and the estimates from four runs were almost identical – the average estimates were 1.0×10^-2^, 8.4×10^-3^  and -2.94 for *H_A_*, *H_Z_* and *log10(ε)*, while the maximum MAD (mean absolute deviation among runs) across the 41 individuals were 3.7×10^-5^, 2.0×10^-4^ and 2.9×10^-3^, respectively. Therefore, the estimates reported and used in downstream analysis were averages from the four independent optimization runs.

We used estimated *H* as an approximation for $\theta$ given:

$$H=\frac{\theta}{1+\theta}\approx\theta$$

when $\theta\ll1$.

With estimated *H* and $\varepsilon$, genotypes were determined by calculating the probabilities of being heterozygous versus homozygous, and SNPs (Single Nucleotide Polymorphisms) were called if the heterozygotes can be assigned with ≥0.95 probability [12].

To minimize the effects of assemble, alignment and mapping errors, we further applied a polymorphism and divergence filter—vetting the mapped RAD loci based on their results from genotype calling, and performed a second round of ML estimation based on filtered data. For each locus, we calculated the percentage of SNPs and the maximum number of SNPs per 10bp, and filtered out the loci with more than 5% variable sites (i.e., possibly a mixture of reads from paralogous gene copies) and loci with more than 4 SNPs segregated in 10bp fragment (possible alignment errors). We also counted the number of fixed differences between the RAD consensus sequences and the zebra finch reference genome, and discarded those loci with more than 20% divergence (possible mapping errors). The amount of data filtered out varies greatly among taxonomic families— 18% (±0.2) for Picidae (woodpeckers and sapsuckers) while only 2.4%(±0.5) for the rest. In Picidae, majority of the RAD loci were filtered due to the 20% divergence cutoff, suggesting difficulties in mapping RAD sequences from distantly related species. Nevertheless, after accounting for phylogenetic distances, dichromatic and monochromatic samples do not differ regards to the percentage of data filtered by this step (linear regression; *p*=0.75). The length of remaining mapped RAD loci is 44,204,263bp (±22,275,246), with no difference regards to dichromatism level (linear regression, *p=* 0.11). The proportion of Z-linked sequences only changed slightly by the filter (5.60% ±0.7%) with little difference between dichromatic versus monochromatic samples (0.14% higher in dichromatic species; *p =* 0.02). The average sequencing coverage is 5.87 (±1.98; linear regression *p* =0.68), almost equal for Z-linked and autosomal loci (0.98±0.03; linear regression *p*=0.71).

In addition to this polymorphism filter, we also applied another six data filtering criterions to test how robust our result is. First, we addressed the issue of slightly higher proportion of Z-linked sequences in dichromatic species by excluding regions on the reference genome that were only mapped by dichromatic or monochromatic samples in a species pair. After this filter, species pairs not only have almost equal percentage of Z-linked sequences (estimated percentage is 0.008% lower in dichromatic samples; *p* =0.83), but also have their estimates of heterozygosities based on the same set of genomic regions. The second filter we applied was a coverage filter. Low sequencing coverage is known to give biased estimates of population genetic diversity [13], but how ratio of genetic diversity would be affected is unknown. Hence, we re-estimated the parameters by only using mapped RAD loci with at least 5x coverage. Lastly, we applied three more stringent divergence cutoffs (i.e., ≤5%, ≤10% and ≤15%). These cutoffs would exclude potential paralogous mapping from the datasets, but also eliminate more variable loci. Last, we only used autosomal loci from Chromosome 1-10. Previous studies have show the avian micro-chromosomes (i.e., chromosome 11-38) have significantly higher rate of sequence evolution as compared to macrochromosomes or intermediate-sized ones [14]. The amount of data for parameter estimation was reduced with additional filters—for example, the 5x coverage filter dramatically reduced the genomic coverage (40 Mbp versus 15 Mbp alignments to reference genome), and we did observe that the estimated genetic diversity decreased as divergence cutoff filters out less conservative loci. Yet, the relative *R_Z:A_* for species pairs (i.e., dichromatic species have higher *R_Z:A_*) are mostly insensitive to these additional data processing steps (Fig. S2). In fact, the estimates of diversity ratio only changed slightly, except for the sapsucker and woodpecker species with low divergence cutoff (the most distantly related family to zebra finch; Fig. S2). Hence, we chose to report the results from the second round of estimation (i.e., minimizing assembly and mapping errors while keeping a larger genomic coverage) in the main text (Fig. 1).

*5. Estimating* substitution rate (µ) — As explained above, SNPs were called for each mapped RAD locus with the estimated genetic diversity (*H*) and sequencing error rate (*ε*). Then, µ can be estimated by simply counting the number of fixed difference between RAD loci and their aligned zebra finch sequences and dividing it by the alignment length (excluding gaps) and the divergent time. For each individual, we separately estimated µ_A_ and µ_z_, and obtained the ratio— µ_z_/ µ_A_.

We also estimate lineage-specific substitution-rate bias. As zebra finch is distantly related to our interested species, majority of the fixed genetic differences might occur on the zebra finch lineage or before the species pair diverged. This might lead to very similar estimates of µ_z_/ µ_A_ for species pairs (i.e., these estimates might have little power to differentiate lineages-specific effects). Hence, we pooled RAD loci across species pairs according to their mapped zebra finch genomic regions. *uclust* was used again to exclude potential paralogous alignments (75% similarity cutoff). In regions where both species have mapped RAD loci, we assigned the substitutions to specific lineage and use these lineage-specific counts to estimate µ_z_/ µ_A_ for the species pairs separately.

For both estimates of µ_z_/ µ_A_, we also examined the effects of different divergence cutoffs (i.e., ≤5%, ≤10% and ≤15%; Fig. S4). Lower cutoffs resulted in lower estimates of substitution rates as well as rate ratio, which is expected when selecting for more conservative loci (i.e., the mutation rates on Z and autosome become more similar; Fig. S4). The pattern that woodpeckers and sapsuckers in general have lower individual estimates (Fig. S4) probably is due to the same reason—only slow evolving RAD loci could be mapped onto a distantly related reference genome. Yet, we did not observe consistently significant difference in substitution rate or substitution-rate ratio between dichromatic and monochromatic species (Fig. S4). In fact, there is only one a significant *p* value (Fig. S4B; ≤20%), but it suggests more severe mutation bias for monochromatic species.

**Fig. S5** *De novo* assembly. Reads were first de-replicated (A) before clustering (B). Dashed lines connect read pairs, which were processed separately for the first two steps. Reads were assigned to putative RAD loci according to the clustering results on both sides (C). In a simple case (Locus 1/1), all read pairs that belonged to one cluster on the left were also grouped together on the right, so they were assigned to one RAD locus. In a more complicated scenario (Locus 2/2 and 2/3), read pairs clustered on one side were separated on the other; so multiple RAD loci were called. That it, every putative RAD locus is a pair of clusters.

**
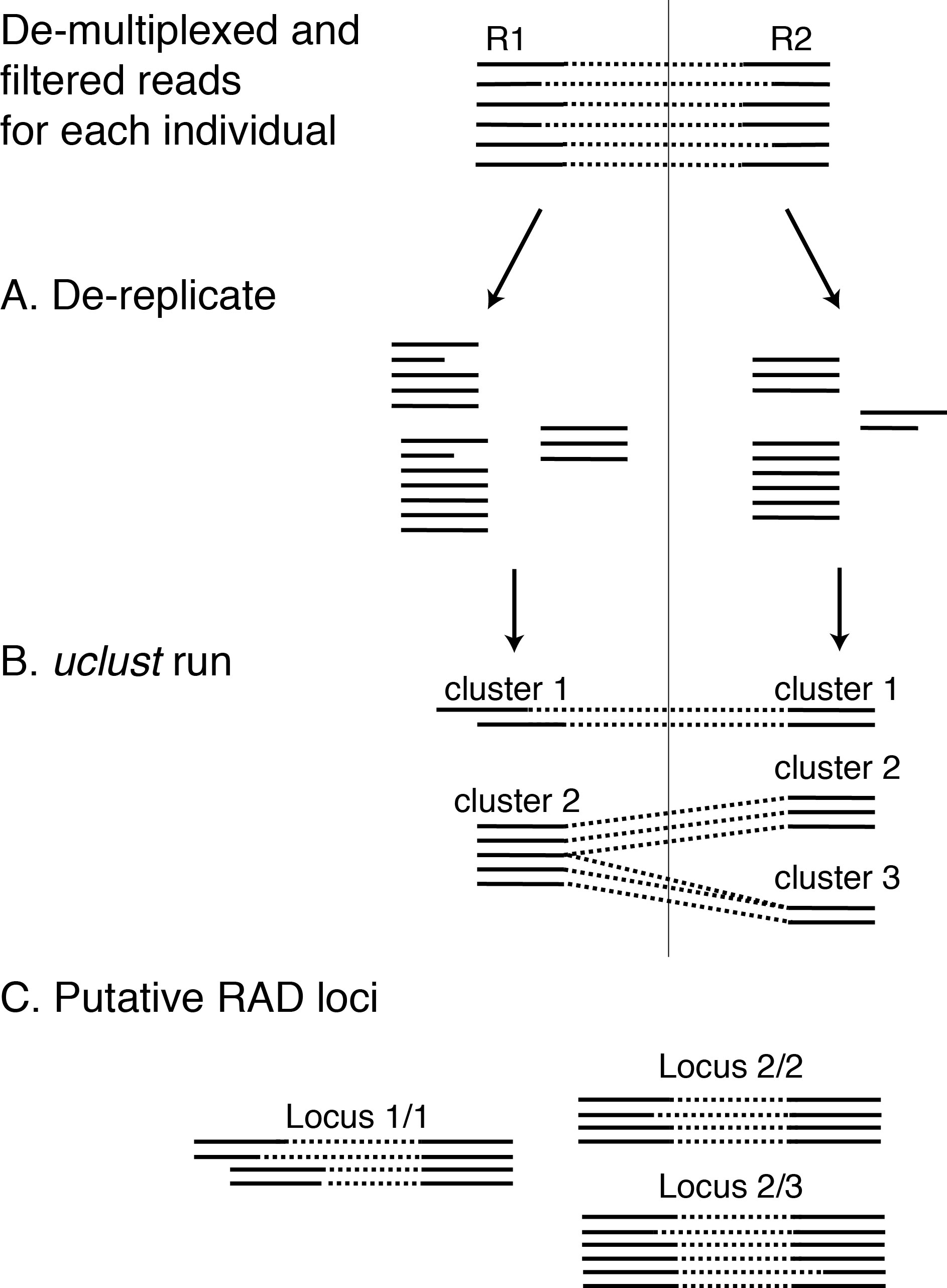
**

**Fig. S6** Diagrams illustrating steps for filtering redundant blasting hits. Thick lines represent the reference genome, and thin lines represent consensus sequences from RAD loci—dark segments indicate aligned sections while grey segments represent un-aligned sections of the sequences. Alignments surrounded by boxes were chosen according to our filtering criteria. For every RAD locus, we first searched for combinations of blast hits that the pair locate within 50kbp range (A), and selected the one with minimal mapping range and *evalue*. If no such combination exists, best hits (i.e., lowest *evalue*) were chosen after excluding alignments shorter than 40bp (B). By calculating the number of aligned RAD loci for every base pair in the zebra finch genome (numbers in top row), a mapped genomic region could be defined as a continuous section of base pairs with non-zero alignments (C). Blast hits that have more than 10bp of unaligned sequences in a region were filtered. If there were more than two clusters among the remaining blast hits, the genomic region was discarded (D). Finally, inclusive sets of genomic regions and RAD loci were identified (arrows connect the RAD loci with genomic regions according to the blasting results), and clusters with multiple or none reference genome sequences were filtered (E).

**
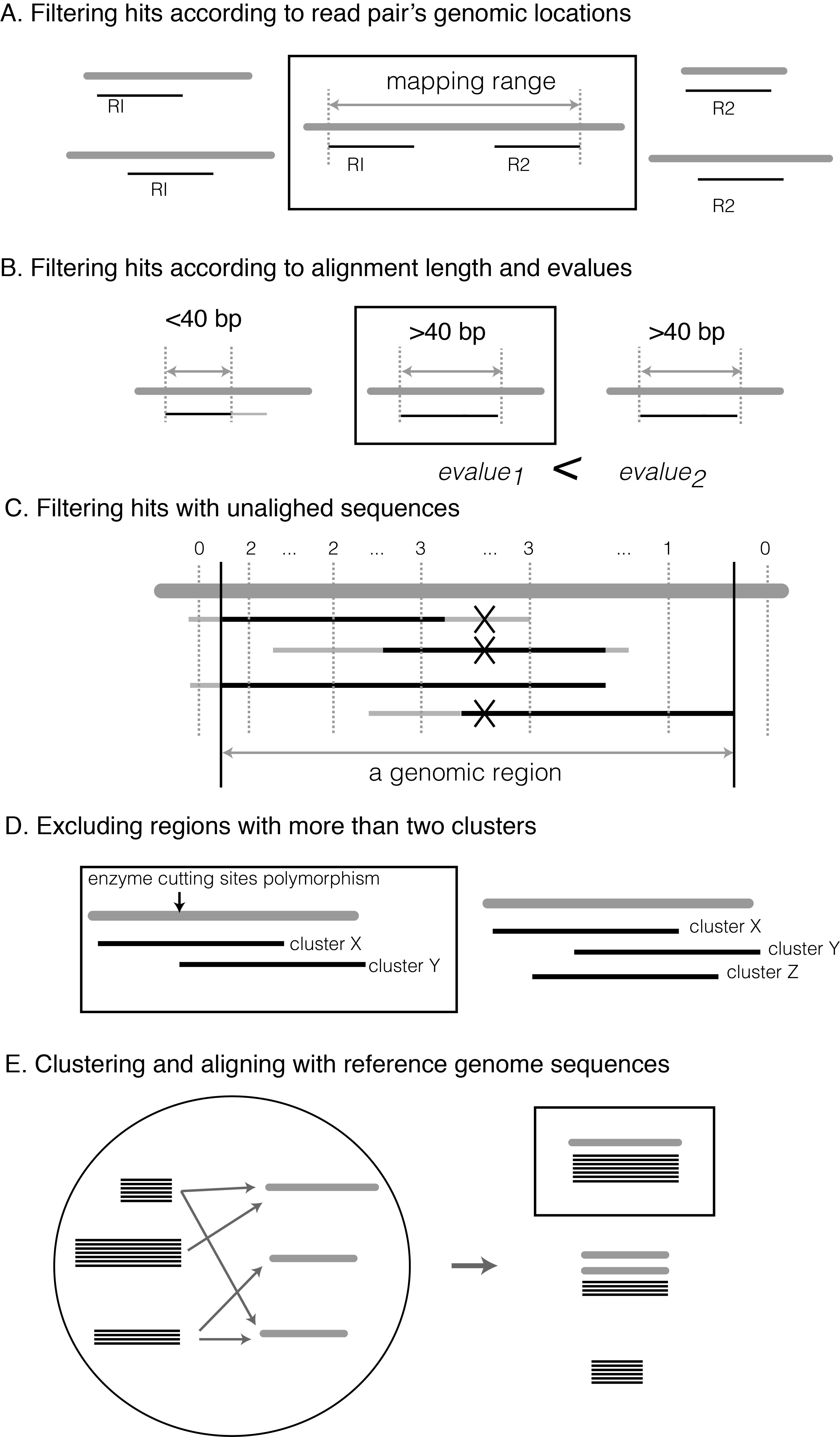
**

**Reference:**

1. Edgar RC: **Search and clustering orders of magnitude faster than BLAST**. *Bioinformatics* 2010, **26**(19):2460-2461.

2. DiBiase A, Harte RA, Zhou Y, Zon L, Kent WJ: **Piloting the zebrafish genome browser**. *Dev Dyn* 2006, **235**(3):747-753.

3. Camacho C, Coulouris G, Avagyan V, Ma N, Papadopoulos J, Bealer K, Madden TL: **BLAST+: architecture and applications**. *BMC Bioinformatics* 2009, **10**:421.

4. **BLAST Command Line Applications User Manual** [<http://www.ncbi.nlm.nih.gov/books/NBK1763/>]

5. Fan WL, Ng CS, Chen CF, Lu MY, Chen YH, Liu CJ, Wu SM, Chen CK, Chen JJ, Mao CT *et al*: **Genome-wide patterns of genetic variation in two domestic chickens**. *Genome Biol Evol* 2013, **5**(7):1376-1392.

6. Meyerhans A, Vartanian JP, Wain-Hobson S: **DNA recombination during PCR**. *Nucleic Acids Res* 1990, **18**(7):1687-1691.

7. Wootton JC, Federhen S: **Analysis of compositionally biased regions in sequence databases**. *Methods Enzymol* 1996, **266**:554-571.

8. Shendure J, Ji H: **Next-generation DNA sequencing**. *Nat Biotechnol* 2008, **26**(10):1135-1145.

9. Lynch M: **Estimation of nucleotide diversity, disequilibrium coefficients, and mutation rates from high-coverage genome-sequencing projects**. *Mol Biol Evol* 2008, **25**(11):2409-2419.

10. Catchen J, Hohenlohe PA, Bassham S, Amores A, Cresko WA: **Stacks: an analysis tool set for population genomics**. *Molecular ecology* 2013, **22**(11):3124-3140.

11. R Development Core Team: **R: A Language and Environment for Statistical Computing**. In*.*; 2008.

12. Li H, Ruan J, Durbin R: **Mapping short DNA sequencing reads and calling variants using mapping quality scores**. *Genome Res* 2008, **18**(11):1851-1858.

13. Han E, Sinsheimer JS, Novembre J: **Characterizing bias in population genetic inferences from low-coverage sequencing data**. *Mol Biol Evol* 2014, **31**(3):723-735.

14. Axelsson E, Webster MT, Smith NGC, Burt DW, Ellegren H: **Comparison of the chicken and turkey genomes reveals a higher rate of nucleotide divergence on microchromosomes than macrochromosomes**. *Genome Res* 2005, **15**(1):120-125.
